# CT-based body composition in diffuse large B cell lymphoma patients: changes after treatment and association with survival

**DOI:** 10.1007/s11547-023-01723-5

**Published:** 2023-09-26

**Authors:** Maria Cristina Pirosa, Fabiana Esposito, Giorgio Raia, Vito Chianca, Andrea Cozzi, Lorenzo Ruinelli, Luca Ceriani, Emanuele Zucca, Filippo Del Grande, Stefania Rizzo

**Affiliations:** 1 Istituto Oncologico Della Svizzera Italiana (IOSI), Ente Ospedaliero Cantonale (EOC), Via Ospedale 1, 6500 Bellinzona, Switzerland; 2 Institute of Oncology Research (IOR), Via Chiesa 5, Bellinzona, Switzerland; 3 Present Address: Istituto Di Imaging Della Svizzera Italiana (IIMSI), Clinica Di Radiologia Ente Ospedaliero Cantonale (EOC), Via Tesserete 46, 6900 Lugano, Switzerland; 4 Policlinico San Donato, Piazza E. Malan 2, 20097 San Donato Milanese, Milan, Italy; 5 ICT (Informatica E Tecnologia Della Comunicazione), Ente Ospedaliero Cantonale, 6500 Bellinzona, Switzerland; 6 CTU (Clinical Trial Unit), Ente Ospedaliero Cantonale, 6500 Bellinzona, Switzerland; 7 Facoltà Di Scienze Biomediche, Università Della Svizzera Italiana (USI), Via Buffi 13, 6900 Lugano, Switzerland

**Keywords:** CT, Body composition, Bone mineral density, Non-hodgkin lymphoma

## Abstract

**Purpose:**

Primary purpose was to assess changes of bone mineral density (BMD) in diffuse large B cell lymphoma (DLBCL) patients treated with rituximab, cyclophosphamide, doxorubicin, vincristine, and prednisone R-CHOP (like) chemotherapy regimen. Secondary purposes were to assess other body composition features changes and to assess the association of pre-therapy values and their changes over time with survival.

**Material and methods:**

Patients selected underwent R-CHOP(like) regimen for DLBCL, and underwent PET-CT before and after treatment. Main clinical data collected included body mass index, date of last follow-up, date of progression, and date of death. From the low-dose CT images, BMD was assessed at the L1 level; the other body composition values, including muscle and fat distribution, were assessed at the L3 level by using a dedicated software. Descriptive statistics were reported as median and interquartile range, or frequencies and percentages. Statistical comparisons of body composition variables between pre- and post-treatment assessments were performed using the Wilcoxon matched pairs signed rank test. Non-normal distribution of variables was tested with the Shapiro–Wilk test. For qualitative variables, the Fisher exact test was used. Log rank test was used to compare survival between different subgroups of the study population defined by specific body composition cutoffs. The significance level was set at *p* < 0.05.

**Results:**

Eighty-two patients were included. The mean follow-up was 37.5 ± 21.4 months. A significant difference was found in mean BMD before and after R-CHOP(like) treatment (*p* < 0.0001). The same trend was observed for mean skeletal muscle area (SMA) (*p* = 0.004) and mean skeletal muscle index (SMI) (*p* = 0.006). No significant association was demonstrated between body composition variables, PFS and OS.

**Conclusion:**

R-CHOP(like) treatment in DLBCL patients was associated with significant reduction of BMD, SMA and SMI.

**Supplementary Information:**

The online version contains supplementary material available at 10.1007/s11547-023-01723-5.

## Introduction

Diffuse large B cell lymphoma (DLBCL) is the most common subtype of non-Hodgkin lymphoma, accounting for approximately 25–35% of adult non-Hodgkin lymphomas in Western countries. It occurs primarily in elderly individuals with a median age of seventy years [1]. Historically, common first-line therapy for DLBCL is based on immunochemotherapy, including rituximab, cyclophosphamide, doxorubicin, vincristine and prednisone, namely referred to as R-CHOP and R-CHOP(like) regimens. Although the addition of R to CHOP has significantly improved treatment outcomes [2], 30–40% of patients are still not cured. During a typical course of treatment, patients receive intermittent doses of prednisone for 5 days in each cycle, up to six cycles within 18 weeks, and these R-CHOP(like) regimens are frequently associated with high incidence of vertebral fractures [3]. Despite these findings, the majority of patients treated with R-CHOP(like) regimens do not receive osteoporosis preventing medication and, to date, there is inadequate information about the risk of osteoporotic fractures in these patients. Moreover, current treatment guidelines, such as the European Society for Medical Oncology and the National Comprehensive Cancer Network, for the treatment of DLBCL [4, 5] neither require pre-treatment bone mineral density (BMD) assessments nor they do implement treatments for accelerated BMD reduction. Oncological patients routinely undergo imaging examinations and these assessments are performed by positron emission tomography computed tomography (PET-CT) in DLBCL patients [4]. CT scans included in PET-CT examinations contain robust additional data on body composition that generally go unused in routine clinical practice [6]. CT images can indeed be used to assess BMD by placing a region of interest (ROI) on the vertebral body of the first lumbar vertebra (L1) [7]. Furthermore, specific software allow the extraction of quantitative measurements from axial CT images, such as skeletal muscle area (SMA), subcutaneous adipose tissue (SAT) and visceral adipose tissue (VAT), usually performed at the level of the third lumbar vertebra (L3), even retrospectively [8–13]. From these values, knowing the height of the patients, it is possible to calculate the skeletal muscle index (SMI = SMA/height^2^).

There are still limited data in the literature about the use of CT to analyze bone loss in terms of vertebral density in DLBCL patients. Few published studies showed a significant reduction of bone mineral density after R-CHOP(like) treatment and common vertebral compression fractures [3; 14]. However, a comprehensive body composition assessment performed by CT scan has been so far under-evaluated in adult NHL patients. Therefore, the primary objective of this study was to assess the association between changes of BMD in DLBCL patients and R-CHOP(like) chemotherapy regimens. Secondary objectives were as follows: the assessment of changes of other body composition values between pre-treatment and post-treatment imaging evaluations, and the association of pre-treatment values and of the value changes after treatment with overall survival (OS) and progression free survival (PFS).

## Methods

### Patient selection

The study population was retrospectively selected from a database of patients with DLBCL referred to our Institution between January 2016 and May 2020, with a minimum follow-up after treatment of 12 months. The Ethics Committee approved this retrospective study with waiver of informed content for deceased patients, and with a no-objection letter sent to the other patients (2022–01111). Inclusion criteria consisted of age ≥ 18 years; histologically proven diagnosis of DLBCL de novo or transformed from follicular lymphoma, R-CHOP like regimen performed as first-line therapy, availability in the picture archiving and communication system of CT images (as part of a PET-CT) before and at the end of treatment, availability of a clinical follow-up > 12 months. Exclusion criteria consisted of technical problems on the images, such as artifacts due to prostheses and impossibility of examination of SMI, VAT and SAT [15], and documented refusal to the use of clinical data for research.

### Clinical data recording

The following clinical data were collected: sex, age at diagnosis, stage, ECOG performance status, calcium, lactate dehydrogenase (LDH), extranodal sites (presence and number), B symptoms, international prognostic index (IPI) score for DLBCL, number of fractures, steroid dose received, radiotherapy, response after first-line therapy, weight and height to calculate the body mass index (BMI, kg/m2), advent of fractures, and date of last contact, date of progression and date of death to calculate OS and PFS, as number of months from the treatment initiation.

### Body composition data extraction

**Fig. 1:**
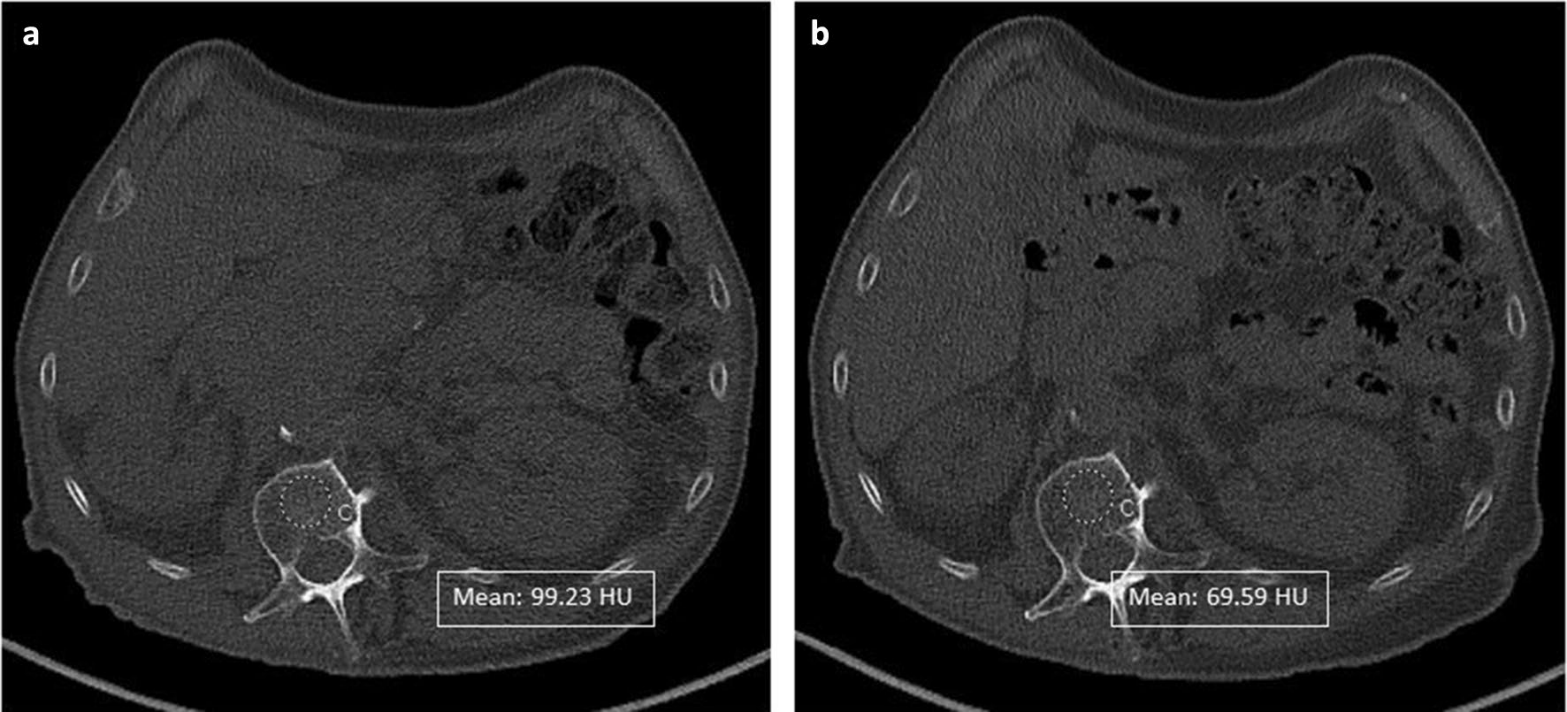
ROI placement at the level of the first lumbar vertebra (L1) in the CT images at baseline **a** and at the end of treatment **b**, showing a reduction in BMD

**Fig. 2:**
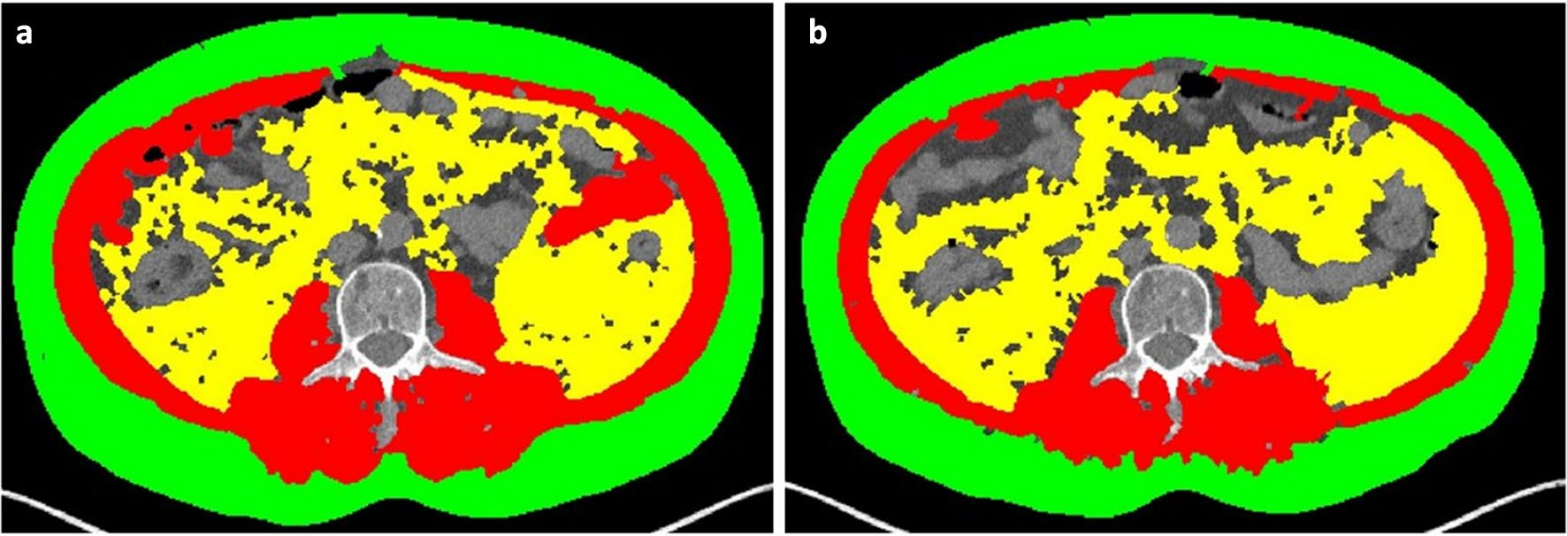
Axial CT images at the level of the third lumbar vertebra (L3), showing automatic segmentation of skeletal muscle area (red), visceral adipose tissue (yellow) and subcutaneous adipose tissue (green) at baseline examination **a** and at the end of treatment **b**, showing a reduction of skeletal muscle area

**Fig. 3:**
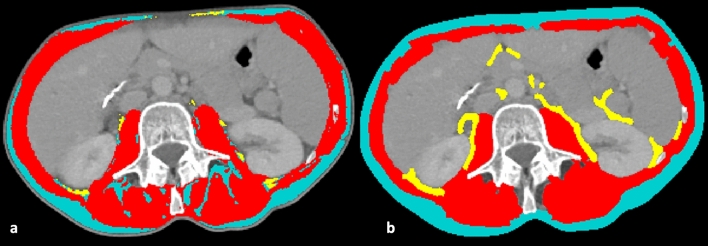
Axial CT image at the level of the third lumbar vertebra (L3), showing segmentation performed by the automatic tool **a**. Since the visceral adipose tissue (yellow) and the skeletal muscle area (red) on the abdominal wall were considered not properly segmented, in this case a semi-automatic segmentation **b** was favored

From PET-CT examinations the low-dose non-contrast phase was used to calculate BMD (measured by Hounsfield Units, HU). The PET-CT examinations were performed on Siemens Somaton Edge 40 slices CT scanner (Siemens Healthineers, Erlangen, Germany). All scans extended in the caudo-cranial direction from the pubis to the neck. Scans were acquired at the following parameters: slice thickness 3 mm, rotation time 0.5 s, tube voltage 120 kV, and tube current automatically adjusted according to CARE Dose 4D (quality ref mAs 30). A dedicated radiologist selected the axial CT image at the L1 level and placed a round region of interest (ROI) in the center of the vertebral body, including the widest area of the trabecular bone, but excluding the cortical bone (Fig. 1). Indeed, it was already demonstrated [77] that an ROI placed in the anterior aspect of the L1 trabecular space on a single axial CT image to measure mean attenuation, requires little training and shows good interobserver agreement [6]. This evaluation was performed directly on the picture archiving and communication system. For the other body composition values, the portal venous phase, when available, was used for segmentations. To this end, an axial CT image at the L3 level was selected and stored in digital imaging and communications in medicine format, and it was then uploaded into the Slice-O-Matic software v 5.0 (Tomovision, Montreal, Canada). Automatic segmentation was performed by using the Automatic Body composition Analyzer using Computed tomography image Segmentation (ABACS) module, commercially available from Voronoi Health Analytics Inc. (Coquitlam, Canada, https://voronoihealthanalytics.com), and integrated as a module into the SliceOmatic software (TomoVision, Magog, Canada, https://tomovision.com) (Fig. 2). In case the automatic tool did not draw a proper segmentation, a CT semi-automatic segmentation was favored and performed by using either the morpho mode or the region growing mode of the Slice-O-Matic software v 5.0 (Tomovision, Montreal, Canada) (Fig. 3). The morpho mode uses mathematical morphology segmentation by computing the watershed of the gradient that gives a mosaic-like appearance to the image. Each region of this mosaic may then be filled with the appropriate tag value, corresponding to the tissue type. The region growing segmentation examines neighboring pixels of initial seed points and determines whether the pixel neighbors should be added to the region. Then, the final step performed by the software is to merge the areas of these regions together, in order to provide a sum of the areas.

At the L3 level, the following quantitative measures, provided by the software used for segmentation, were recorded: SMA (including the psoas, erector spinae, quadratus lumborum, transversus abdominis, external obliques, internal obliques, and rectus abdominis muscles) expressed in cm^2^, SAT expressed in cm^2^, and VAT expressed in cm^2^. The lumbar SMI was then calculated by dividing the SMA by the height squared (m^2^) and reported as cm^2^/m^2^.

### Statistical Analysis

A preliminary analysis to select a proper sample size was performed, referring to published data [3]. For a normal distribution of BMD and a power of 0.85, with alpha = 0.05, the total sample size should have been n = 84 (Wilcoxon signed rank test matched pairs) [16]. Data from clinical records were summarized in an Excel file (Microsoft Corporation, Redmond, WA, USA). Descriptive statistics were reported as median and IQR, or frequencies and percentages. After ascertaining the distribution of quantitative variables through the Shapiro–Wilk test, statistical comparisons of body composition variables between pre- and post-treatment assessments were performed using the Wilcoxon matched pairs signed rank test. For qualitative variables, the Fisher exact test was used. Log rank test was used to compare survival between different subgroups of the study population defined by specific body composition cutoffs. For sarcopenia, the sex-specific cutoffs were SMI < 43 for men with BMI < 24.9 kg/m^2^, and SMI < 53 for men with BMI > 25 kg/m^2^, whereas it was SMI < 41 for women of any BMI [17]. For the other body composition values, the median of each variable was set as the cutoff value. The univariate Cox regression model was used to study independent associations of body composition parameters. The significance level was set at p < 0.05. Statistical analyses were performed using STATA17 (StataCorp., College Station, TX, USA).

## Results

Main population characteristics are summarized in Table 1. Eighty-two patients (34 women and 48 men, median age 71.5, age range 60–78 years) were included in the study. Most patients (61%) were at stage IV and had an ECOG score of 1 at diagnosis (58.5%). Extranodal sites were found in 75.6% of patients, mainly involving bone, spleen, and stomach, with a mean number of extranodal sites per patient of 1.8 ± 1.2. Approximately 40% of patients had an IPI score of 1 or 2, while 35.4% had an IPI score of 3. Fractures during the follow-up period occurred in 5 patients (6%). Mean steroid dose received was 2631 ± 937 mg (range 450–5760 mg); 35.4% of patients received radiotherapy. After first-line therapy, 69.5% of patients achieved a complete response, 8.5% showed a partial response, and 22% showed disease progression. Twenty-three patients underwent a second cycle of treatment. Overall, disease progression was observed in 27 patients (32.9%). At the end of the study, 23 patients had died. The mean follow-up time was 37.5 ± 21.4 months.

**Table 1:** Main patient characteristics

No. of patients	82	
*Gender, n (%)*		
Female	34	(41.5%)
Male	48	(58.5%)
*Age*		
Mean (SD)	68.8	(13.7)
Range	20	93
Median (25^th^ – 75^th^)	71.5	(60–78)
*Stage, n (%)*		
I	10	(12.2%)
II	11	(13.4%)
III	11	(13.4%)
IV	50	(61.0%)
*ECOG, n (%)*		
0	20	(24.4%)
1	48	(58.5%)
2	14	(17.1%)
Calcium (mmol/L), mean (SD)	2.3	(0.3)
Median (IQR)	2.3 (2.2–2.4)	
LDH (mU/mL), mean (SD)	640.3	(874.4)
Median (IQR)	441.0 (352.0–683.)	
Extranodal site, n (%)	62	(75.6%)
Number of extranodal sites, mean (SD)	1.8	(1.2)
B Symptoms, n (%)	15	(18.3%)
*IPI Score, n (%)*		
0	5	(6.1%)
1	16	(19.5%)
2	17	(20.7%)
3	29	(35.4%)
4	14	(17.1%)
5	1	(1.2%)
Fractures	5	(6%)
Mean steroid dose mg (SD)	2631	(937)
Radiotherapy, n (%)	29	(35.4%)
*Response after 1 line therapy, n (%)*		
Complete Response	57	(69.5%)
Disease Progression	18	(22.0%)
Partial Response	7	(8.5%)
Therapy after 1^st^ line, n (%)	23	(28.1%)
*Outcome*		
Follow-up (months), mean (SD)	37.5	(21.4)
Median (IQR)	35.5 (19–58)	
Progression Disease	27	(32.9%)
Death, n (%)	23	(28.1%)
Progression Free Survival (min–max)	2–74 months	
Overall Survival (min–max)	1–75 months	

The mean BMI was 25.31 ± 5.67; patients with BMI < 18.5 were 4 (4.88%); patients with BMI between 18.5 and 24.9 were 41 (50%); patients with BMI between 25.0 and 29.9 were 24 (29.27%); patients with BMI ≥ 30.0 were 13 (15.85%).

**Fig. 4:**
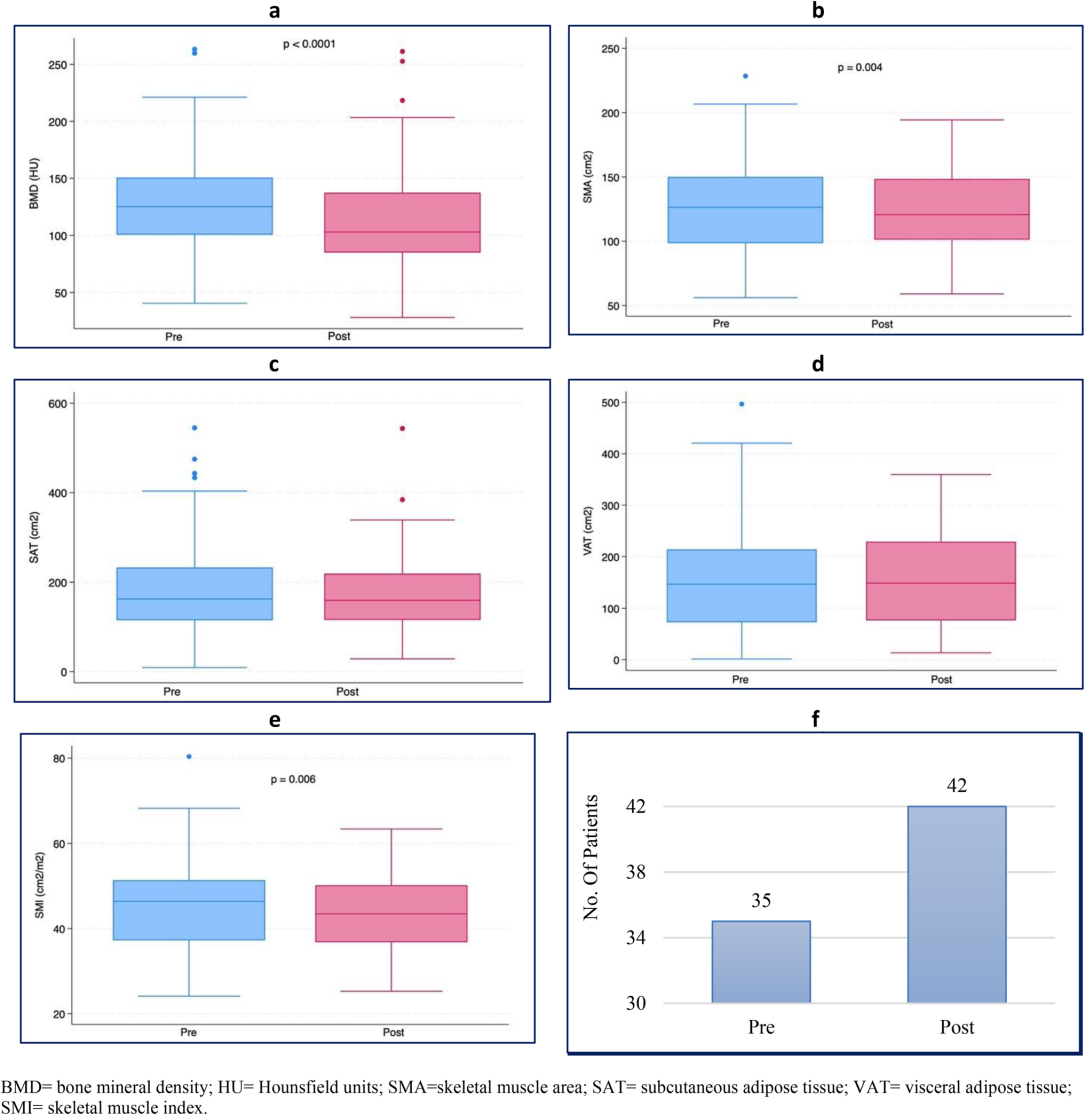
Pre- and post-treatment **a** BMD; **b** SMA, **c** SAT, **d** VAT, **e** SMI, **f** Number of sarcopenic patients (Cutoff defined according to [21]) before and after therapy. *P* value was determined through the Wilcoxon matched pairs signed rank test for **a**, **b**, **c**, **d** and **e** comparisons, while it was determined through Fisher exact test for **f**

Post-treatment BMD (median: 102.97 HU, IQR: 85.0–137.4 HU) was significantly lower than pre-treatment BMD (median: 125.2 HU, IQR: 100.6–150.7 HU) (*p* < 0.0001). The same trend of BMD was observed for SMA (pre-treatment: median: 126.4 cm^2^, IQR: 98.6–150.0 cm^2^; after-treatment: 120.7 cm^2^, IQR: 101.2–148.4 cm^2^; *p* = 0.004) (Fig. 2) and SMI (pre-treatment: 46.4 cm^2^/m^2^, IQR: 37.2–51.3 cm^2^/m^2^; after-treatment: 43.5 cm^2^/m^2^, IQR: 36.8–50.2 cm^2^/m^2^; *p* = 0.006) (Fig. 4 and Supplementary table 1). No significant changes were seen for SAT and VAT. Approximately 43% of patients were sarcopenic before treatment, and 51.2% were sarcopenic after treatment (*p* < 0.001).

**Fig. 5:**
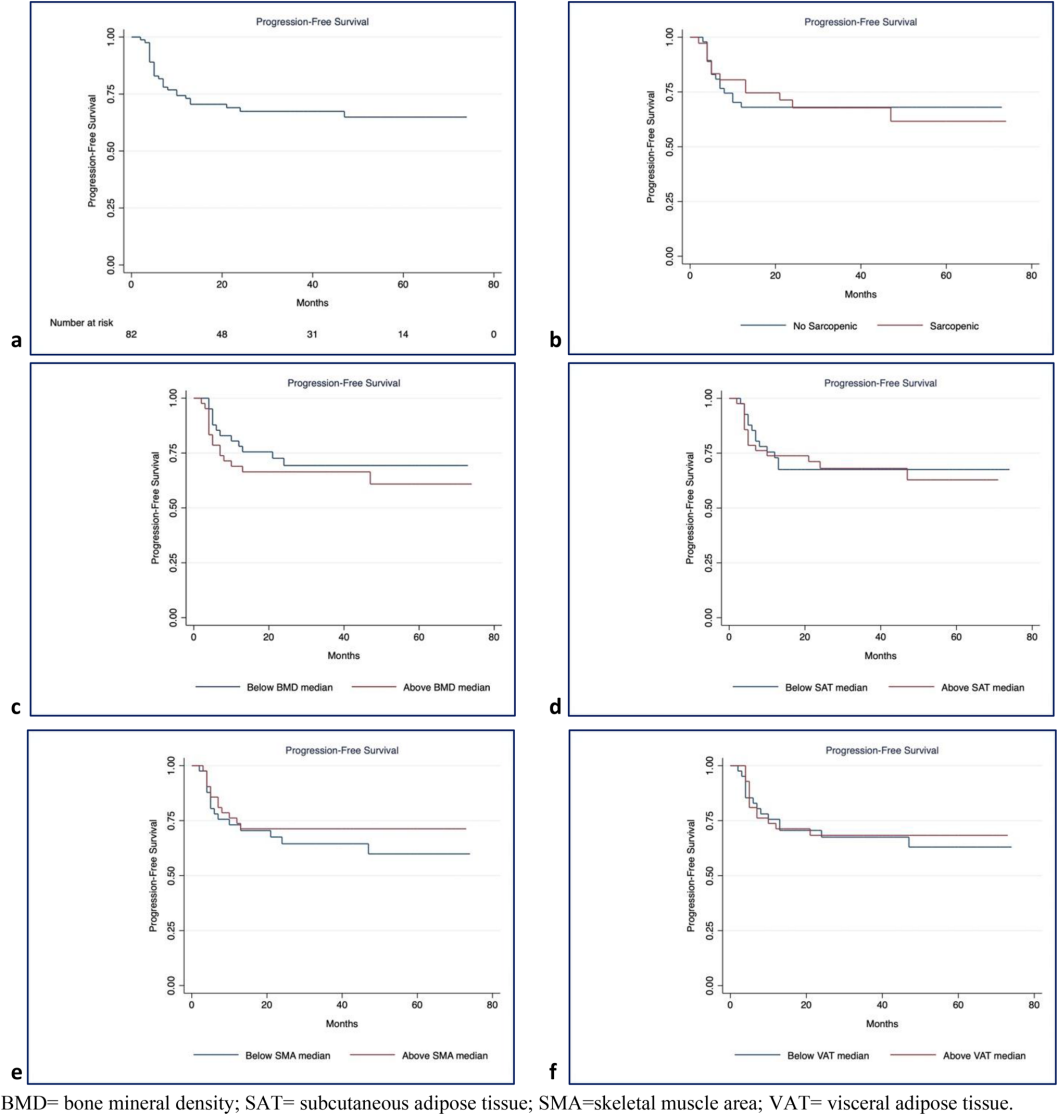
Kaplan–Meier curves of Progression Free Survival. **a** progression free survival for the whole cohort. Influence on progression free survival of: **b** Sarcopenia (chi2(1) = 0.02, *p* = 0.898), **c** BMD (chi2(1) = 0.79, p = 0.373), **d** SAT (chi2(1) = 0.05, *p* = 0.820), **e** SMA (chi2(1) = 0.42, *p* = 0.5194), **f** VAT (chi2(1) = 0.03, *p* = 0.8640)

**Fig. 6:**
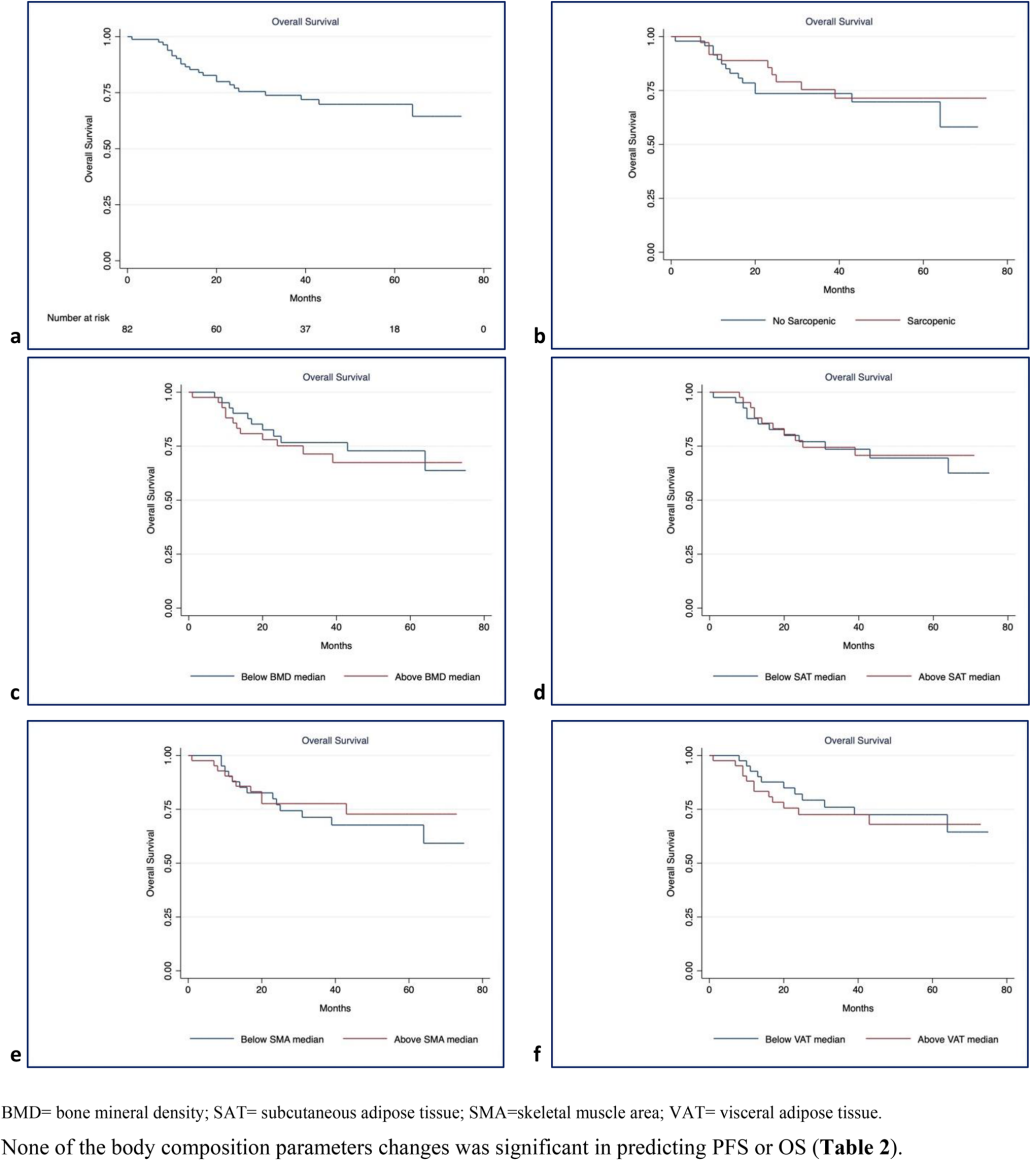
Kaplan–Meier curves of Overall Survival. **a** Overall survival for the whole cohort. Influence on overall survival of **b** Sarcopenia (chi2(1) = 0.27, p = 0.600), **c** BMD (chi2(1) = 0.24, p = 0.6268), **d** SAT (chi2(1) = 0.01, *p* = 0.9198), **e** SMA (chi2(1) = 0.21, *p* = 0.6488), **e** VAT (chi2(1) = 0.28, *p* = 0.5986).

Survival plots for PFS and OS using the Kaplan–Meier method showed rapid disease progression in the first 24 months for PFS (survival function: 0.68, SE: 0.05, 95%CI: 0.56; 0.77) and stabilization in the following months. A similar trend with a lower slope was observed for OS, for which the survival function in the first 31 months was 0.74 (SE: 0.05, 95%CI: 0.63; 0.83). When the influence of body composition parameters before treatment on PFS (Fig. 5) and OS (Fig. 6) was analyzed, there were no differences in the log-rank tests for all comparisons.

None of the body composition parameters changes was significant in predicting PFS or OS (Table 2).

**Table 2:** Univariate Cox’s regression model showing that none of the body composition parameters was statistically significant in predicting PFS or OS

	Progression free survival		Overall survival	
	HR (SE)	95%CI	HR (SE)	95%CI
BMD post—BMD Pre	0.98 (0.01)	0.98–1.02	1.01 (0.01)	0.99–1.04
SMA post—SMA pre	1.00 (0.01)	0.98–1.02	1.01 (0.01)	0.98–1.04
VAT post—VAT pre	1.00 (0.01)	0.99–1.01	1.00 (0.01)	0.99–1.00
SAT Post—SAT pre	1.00 (0.01)	1.00–1.01	1.00 (0.01)	0.99–1.01
SMI post—SMI pre	1.00 (0.03)	0.94–1.07	1.03 (0.04)	0.95–1.11

## Discussion

In our single-center study on a cohort of 82 patients with newly diagnosed DLBCL, we demonstrated a significant difference in BMD measured by CT before and after treatment with a combination of R-CHOP(like) chemotherapy regimens and steroids, although only 5/82 (6%) patients reported fractures during the period evaluated.

The literature about the use of CT scan to assess the bone loss in terms of vertebral density in DLBCL patients is still limited. However, the high incidence of osteoporosis and fractures in patients undergoing chemotherapy regimen with steroids is well known [18] and multifactorial [3, 19–22]. The specific population of patients with DLBCL receives intermittent doses of chemotherapy for a limited number of days during a median period of 6 months, as well as high dose of steroids. Moreover, in these patients, there may be an altered bone metabolism and changes of muscle mass due to the release of inflammatory cytokines [18, 23, 24].

Our results are concordant with the recently published results of patients with DLBCL treated with R-CHOP(like) in the FLYER trial, where a post hoc analysis comparing four cycles of R-CHOP + 2 × rituximab with a regimen of six cycles of R-CHOP in young and favorable DLBCL patients, and showed that four cycles of R-CHOP were associated with less BMD loss than patients receiving six cycles [25]. Our results, by capturing the presence of significant variations in BMD in DLCBL patients, highlight the interesting possibility to assess BMD from an imaging examination that is performed as a routine exam, thus offering helpful information about the bone density status in DLBCL patients with no adjunctive costs. Furthermore, with BMD normative values at CT available [7], this quantitative evaluation may in the future avoid to perform a dual energy X-ray absorptiometry (DEXA) in DLBCL patients after treatment.

In our cohort, we demonstrated also a significant reduction of SMA and SMI, as well as significant increase in sarcopenic patients, after treatment. These results are concordant with a large study examining short- and long-term body composition changes in DLBCL survivors following R-CHOP based chemotherapy [26]. Indeed, in a subgroup of 191 DLBCL patients, the authors demonstrated a significant SMA reduction of 2.8% after completion of treatment. On the other hand, the same authors demonstrated a significant increase in SAT and VAT from baseline (+ 6.5% and + 4.5%, respectively) that was not confirmed in our patients. However, there are differences in the body composition of their population compared to our study population, since their cohort included white and black patients from the US, with a 79.3% of sarcopenic patients before treatment and 20.7% with newly identified sarcopenia after treatment; conversely, in our population, we found 43% of patients sarcopenic before treatment and 51.2% sarcopenic after treatment (*p* < 0.001) [26]. Moreover, in their cohort, 30.4% of patients showed a baseline BMI higher than 30 kg/m^2^ [26], whereas in our cohort only 15% falled in this BMI category.

In another study performed on 138 non-Hodgkin lymphoma patients, including different histological types and different treatment regimens, among the strongest prognostic factors there were sarcopenia, evaluated by psoas muscle mass index, psoas area, psoas density (*p* < 0.01), and adipopenia, evaluated by SAT (*p* < 0.01) [31]. In our study, although the significant changes of SMA and SMI, neither these parameters nor the adipose tissue parameters (SAT and VAT) were associated with prognosis in terms of OS and PFS [23]. However, compared to De Guevara et al., our patients selection was more restrictive, including only one type of non-Hodgkin lymphoma (DLBCL) and one type of therapy regimen, in order to make more clean comparisons, not influenced by histology and chemotherapy. A Chinese retrospective study on 201 DLBCL patients evaluated the correlation between CT-based body composition parameters with treatment-related toxicity and other adverse outcomes. The authors demonstrated that skeletal muscle density (SMD), SMI, skeletal muscle gauge (SMIxSMD), and lean body mass correlated with any grade 3–4 toxicity, dose reduction and hospitalization [28]*.* On the other hand, the authors demonstrated a significant association between SMI and PFS [30], that is not concordant with our results. This discrepancy may be explained by the different cutoffs used by the authors (27.55 for SMI), compared to our cutoffs, referring to Martin et al. [17].

Unfortunately, the inconsistency of cutoff values for SMI, sarcopenia and other body composition parameters is a known problem to allow comparisons of body composition assessments, and it has been described by other recent studies [9, 11].

In our cohort, the body composition metrics related to the fat distribution (SAT and VAT) did not demonstrate any significant change after treatment, nor was related to survival. Accordingly, another study performed on 118 DLBCL patients treated by immune-chemotherapy showed no significant change in minimal perirenal fat, maximal perirenal fat, nor subcutaneous fat thicknesses at the end of treatment [29]. Interestingly, Xiao et al. demonstrated that 24 months after treatment with R-CHOP(like) regimen, DLBCL patients gained weight mainly by gaining fat, instead of by gaining muscle tissue [26], thus confirming that DLBCL patients are at risk for a short-term and long-term development of body composition changes.

The present study has some limitations. The first is related to its retrospective nature combined with the monocentric approach that may have led to selection bias. Furthermore, we included an age-unhomogeneous population in the study, with a median age of 71.5 years (range 60–78). In the elderly, in fact, the etiology of osteoporosis may be aggravated by other mechanisms such as the reduction of sex hormones (androgens, estrogen) and muscle mass [24]. In addition, the sample size can be small to draw conclusions that can be generalizable. Therefore, our results might deserve validation by a prospective study with a larger population including also young adults. Moreover, for assessment of BMD, we used the low-dose CT scan of the PET-CT, for consistent comparisons, because this series was present in all the scans. Nevertheless, measurement of HU on low-dose CT scans has been demonstrated as a reliable method to assess two-year changes in trabecular bone density at each vertebra from C3-L5 [30], especially if it is intended for the local assessment of trabecular bone changes and not for the clinical diagnosis of osteoporosis, as in our study [31]. Another limitation is that we did not assess if the reduction of BMD, SMA and SMI was associated with clinical effects different from OS and PFS, such as pain, fatigue, metabolic disease. However, given the retrospective nature of the study, it was not possible to objectivate the patients quality of life at the time of the imaging examination. Finally, we did not assess the presence of associations with the density of muscle, which may be considered an indicator of the muscle quality (instead of quantity), because it indicates the fat infiltration within the muscle area. However, to ensure consistent intra-patients comparisons, we decided to exclude a priori this assessment because it relies on the HU units, that is highly affected by the presence of contrast medium that was not always present in the PET-CT examinations included in this study.

In conclusion, this study has demonstrated that R-CHOP(like) treatment in DLBCL patients is associated with a significant reduction of BMD, SMA and SMI. On the other hand, the assessed body composition parameters changes were not associated with OS and PFS, whereas association with other quality of life parameters was not assessed. As radiology transitions from a volume-based to a value-based practice, CT opportunistic screening of body composition assessment based on CT studies, routinely performed by many oncological patients, including DLBCL patients, may add value to examinations that are already provided as standard of care, thus eventually offering the possibility to use available information that currently goes unused, to guide individualized lifestyle interventions.

### Supplementary Information

Below is the link to the electronic supplementary material.

Supplementary file1 (DOCX 14 KB)

## Abbreviations

BMI: Body mass index
BMD: Bone mineral density
DLBCL: Diffuse large B cell lymphoma
ECOG: Eastern cooperative oncology group
L1: First lumbar vertebra
L3: Third lumbar vertebra
OS: Overall survival
PFS: Progression free survival
PET-CT: Positron emission tomography computed tomography
R-CHOP: Rituximab, cyclophosphamide, doxorubicin, vincristine, prednisone
ROI: Region of interest
SAT: Subcutaneous adipose tissue
SMA: Skeletal muscle area
SMI: Skeletal muscle index
VAT: Visceral adipose tissue
