# Supplementary material for: CT-based body composition in diffuse large B cell lymphoma patients: changes after treatment and association with survival
Source: Radiol Med. 2023 Sep 26;128(12):1497–507. doi: 10.1007/s11547-023-01723-5 (PMC10700208; doi:10.1007/s11547-023-01723-5)
Supplement: Supplementary file 1 — Supplementary file1 (DOCX 14 KB) [file 11547_2023_1723_MOESM1_ESM.docx]

**Supplementary table 1. Pre- and post-therapy values of body composition values, with the respective p-value.**

|  | Pre | | Post | | p-value |
| --- | --- | --- | --- | --- | --- |
| BMD, median (IQR) | 125.2 | (100.6-150.7) | 103.0 | (85.0-137.4) | < 0.001 |
| SMA, median (IQR) | 126.4 | (98.6-150.0) | 120.7 | (101.2-148.4) | 0.004 |
| SAT, median (IQR) | 162.7 | (115.1-232.5) | 159.7 | (115.9-218.9) | 0.315 |
| VAT, median (IQR) | 147.0 | (72.8-214.0) | 148.9 | (76.7-228.9) | 0.441 |
| SMI, median (IQR) | 46.4 | (37.2-51.3) | 43.5 | (36.8-50.2) | 0.006 |
| Sarcopenia, n (%) | 35 | (42.7%) | 42 | (51.2%) | 0.425 |

BMD= bone mineral density; SMA=skeletal muscle area; SAT= subcutaneous adipose tissue; VAT= visceral adipose tissue; SMI= skeletal muscle index; sarcopenia defined according to [17].
